# Modulating Attentional Load Affects Numerosity Estimation: Evidence against a Pre-Attentive Subitizing Mechanism

**DOI:** 10.1371/journal.pone.0003269

**Published:** 2008-09-24

**Authors:** Petra Vetter, Brian Butterworth, Bahador Bahrami

**Affiliations:** 1 Institute of Cognitive Neuroscience, University College London, London, United Kingdom; 2 Department of Psychology, University College London, London, United Kingdom; Lund University, Sweden

## Abstract

Traditionally, the visual enumeration of a small number of items (1 to about 4), referred to as subitizing, has been thought of as a parallel and pre-attentive process and functionally different from the serial attentive enumeration of larger numerosities. We tested this hypothesis by employing a dual task paradigm that systematically manipulated the attentional resources available to an enumeration task. Enumeration accuracy for small numerosities was severely decreased as more attentional resources were taken away from the numerical task, challenging the traditionally held notion of subitizing as a pre-attentive, capacity-independent process. Judgement of larger numerosities was also affected by dual task conditions and attentional load. These results challenge the proposal that small numerosities are enumerated by a mechanism separate from large numerosities and support the idea of a single, attention-demanding enumeration mechanism.

## Introduction

Jevons found that he could estimate the number of beans in a box without error when there were four or fewer, but became increasingly inaccurate as the number of beans increased beyond four [1]. Subsequent studies have confirmed his findings, and it is now generally assumed that the immediate and accurate apprehension of the numerosity of collections of four or fewer objects uses a process separate from enumerating larger collections [2]–[6]. Following Kaufman and colleagues, this process is called “subitizing” [7].

The current basis for this distinction has come from a discontinuity in the slope of the curve that relates enumeration time to the number of items to be enumerated. Enumeration in the “subitizing range” (1 to 3 or 4 items) typically yields a shallow slope whereas the slope for 5 items and above (the “counting range”) is considerable steeper. This pattern has traditionally been fitted with a bilinear function and two functionally separate enumeration mechanisms have been inferred (see [2] for a review). Furthermore, by analogy with classical studies of visual search [8], a parallel and pre-attentive process has been inferred from the shallow slope for subitizing (equivalent to pop-out search) and a serial and attentive process from the steeper slope (equivalent to conjunction search) for counting [3], [9].

Support for this distinction has come from brain imaging studies that show quantitative differences in parietal lobe activity for the counting range as compared with the subitizing range [4], [6]. More specific evidence for a pre-attentive subitizing mechanism has come from a neuropsychological study of neglect patients [10]. Neglect patients with extinction, who cannot report items in the contra-lesional field due to their inability to attend to this side of space, can nevertheless enumerate up to four objects when two of them are in the neglected field [10].

However, one brain-imaging study has failed to distinguish between the neural substrates of subitizing and counting, and found instead that human parietal cortex activation increased linearly with the number of items [11]. Balakrishnan and Ashby questioned the basis of the initial inference of two mechanisms from the performance data by demonstrating that a bilinear fit is unjustified and a continuous model of enumeration is equally supported by the performance data [12], [13].

Moreover, the strong notion of pre-attentive/attentive dichotomy has been regarded as an oversimplified account in the attention literature (e.g. [14], [15]) and particularly the hypothesis of attention-free perceptual processing has been questioned [16], [17]. Indeed there is evidence that even the simplest forms of feature detection (e.g. orientation detection), which had previously been thought of as occurring pre-attentively, depend on the availability of attentional resources in a dual-task situation [18].

In this study, we investigated how the judgement of both small and large numerosities is affected by a withdrawal of attentional resources, and more specifically, we tested the hypothesis that subitizing is a pre-attentive process. We reasoned that if subitizing is pre-attentive, it should be unaffected by experimental manipulations such as dual-task paradigms that reduce the availability of attentional resources [16], [18]. In addition to imposing an additional task onto a numerosity judgement task, we employed the framework of load theory [19], [20]. Load theory states that in a dual task situation, processing of secondary task stimuli depends on the attentional requirements of the primary task. Under high attentional load, processing capacity is entirely dedicated to the primary task leading to reduced (and sometimes eliminated) processing of the secondary task. Under low attentional load, however, the capacity limit is not reached and attentional resources “spill over” to perform the secondary task.

In this experiment, we combined a secondary numerosity judgement task with a primary task with two levels of attentional load (low and high load). We predicted that if subitizing is a pre-attentive process, it should not be affected by dual versus single task manipulations and, more importantly, subitizing should not be affected by attentional load. However, if subitizing is constrained by attentional capacity, it should be compromised by both experimental manipulations.

## Methods

### Subjects

14 subjects (mean age: 23.1, 10 females) with normal or corrected-to-normal vision participated. All gave written informed consent and were paid for their participation. The study was approved by the ethics committee of the Dept. of Psychology at UCL.

### Visual stimulus

**Figure 1:**
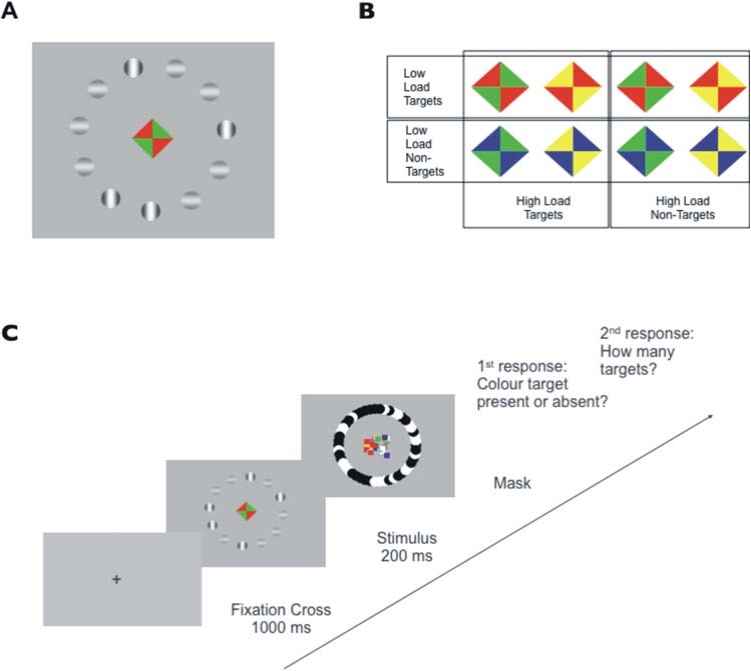
Stimuli and Experimental Procedure. (a) Stimulus example. As primary task, subjects detected a certain colour target at fovea. As secondary task, subjects judged the numerosity of high-contrast gabor patches (1 up to 8) amongst low-contrast distractors. (b) Colour combinations of the primary task. Under low attentional load, detection of a single feature was required (the colour red). Under high attentional load, subjects detected specific conjunctions of colour and spatial arrangement (either green triangles aligned along the right-tilted diagonal or yellow triangles along the left-tilted diagonal). (c) Experimental procedure. Under dual task conditions, subjects responded first to the primary task and subsequently to the secondary task. Under single task conditions, subjects responded only to one of the tasks and ignored the stimuli of the other task.

The visual stimulus consisted of: (i) a central diamond shape (4° of visual angle) comprising 4 coloured triangles and (ii) a circle of gabor patches (10°) on a grey background (see example stimulus in Fig. 1a). Eight different colour combinations were used for the central diamond shape (Fig. 1b). The gabor patches (2° each) in the circle were either vertically oriented high-contrast (100%) targets or horizontally orientated low-contrast (50%) distractors. The distance between patches was equal, patches occupied a different position in the circle in each trial and positions of targets and distractors within the circle were randomly assigned. The grey value of the background was adjusted to mid-grey and gamma corrected for output luminance (as was the gabor value). Stimuli were generated using the Cogent toolbox (www.vislab.ucl.ac.uk/Cogent/) for MATLAB (Mathworks, Inc).

### Task and experimental procedure

We employed a dual task paradigm. The primary task was a speeded target detection task at fovea which implemented the manipulation of attentional load. Under low load, subjects detected a simple feature (the colour red, independent of spatial arrangement), whereas under high load, subjects detected specific conjunctions of colour and spatial arrangement: either two green triangles aligned along the right-tilted diagonal or two yellow triangles aligned along the left-tilted diagonal (see Fig 1b). Importantly, subjects were instructed not to respond to the opposite combinations. Both low and high load condition consisted of the same set of stimuli, only the task instructions changed.

As a secondary task, subjects judged the number of targets ranging from 1 to 8. Total number of items in the circle ranged from 9 to13, counterbalanced for each target number and load condition. Distractors were used to de-correlate task difficulty from the overall processing effort required for multiple stimuli. The number of distractors did not co-vary with the number of targets. Therefore, numerosity judgement could be made neither on the basis of the total number of items present nor on the basis of the number of distractors. As distractors were equally luminous than targets, numerosity could not be judged based on overall luminance either.

After a fixation cross (1s), the stimulus was displayed for 200 ms, followed by a mask which stayed on the screen until subjects responded (Fig. 1c). Inter-trial intervals varied randomly between 1 and 2 seconds. Note that short stimulus durations prevented verbal counting.

Subjects always responded first to the primary task and subsequently to the secondary task, ensuring that attentional resources were manipulated by the processing requirements of the primary task and not by the number of items in the secondary task. Subjects responded with their right hand on two adjacent keys to the primary task and with their left hand to the secondary task using number keys 1–8.

Overall, accuracy was emphasised over speed. Subjects were given practice trials before each block and had the opportunity to take breaks. The testing session lasted 1h.

### Experimental design

Each colour combination of the primary task was combined once with each target numerosity of the secondary task, resulting in 64 trials per block.

Subjects first performed 2 blocks of each task under single task condition (1 block low load, 1 block high load). Subjects were therefore well trained in each of the two tasks before being tested under dual task conditions. 4 blocks of dual task were performed (2 low and 2 high load in the order ABBA or BAAB, counterbalanced across subjects). Each subject performed 16 trials per target number per experimental condition (512 trials for the whole experiment).

## Results

### Primary task–Load manipulation (Fig. 2)

Reaction time and accuracy data of the primary task were compared using a repeated measures ANOVA with within subject factors “load condition” (low load vs. high load) and “task” (single task vs. dual task). As expected, subjects responded more slowly under high attentional load compared to low attentional load (*F* (1,13) = 114.57, *p*<.001) and significantly less accurately (*F* (1,13) = 20.26, *p* = .001 ). Subjects were also slower under dual-task conditions (*F*(1,13) = 97.77, *p*<.001) and less accurate (*F*(1,13) = 37.01, *p*<.001) compared to single task conditions. These results confirm that our manipulation of attentional load was effective.

**Figure 2:**
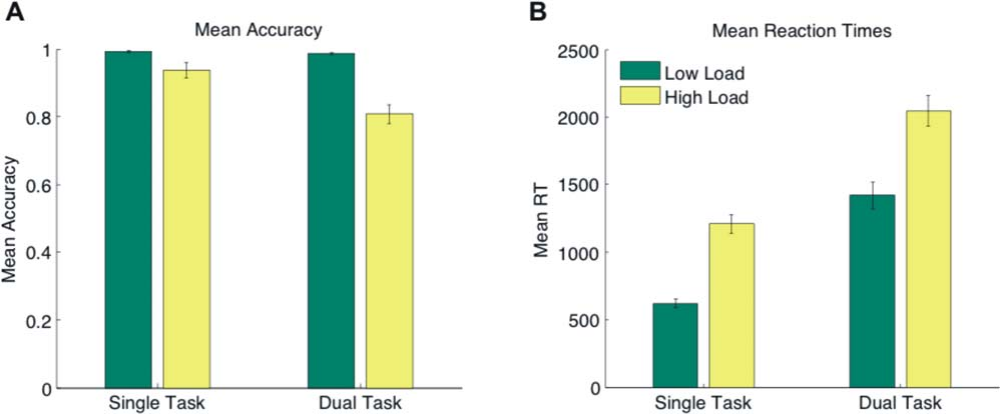
Results of the Colour Detection Task. (a) Mean accuracy (proportion correct) and (b) mean reaction times (ms) of the primary (colour detection) task under single task and dual task conditions (low load: green bars, high load: yellow bars). Error bars indicate one standard error of the mean.

### Secondary task–Numerosity judgement

#### Accuracy (Fig. 3a)

Due to the sequential key responses to the primary and secondary task, reaction time data of the secondary (numerosity) task was not very meaningful and is not reported here.

**Figure 3:**
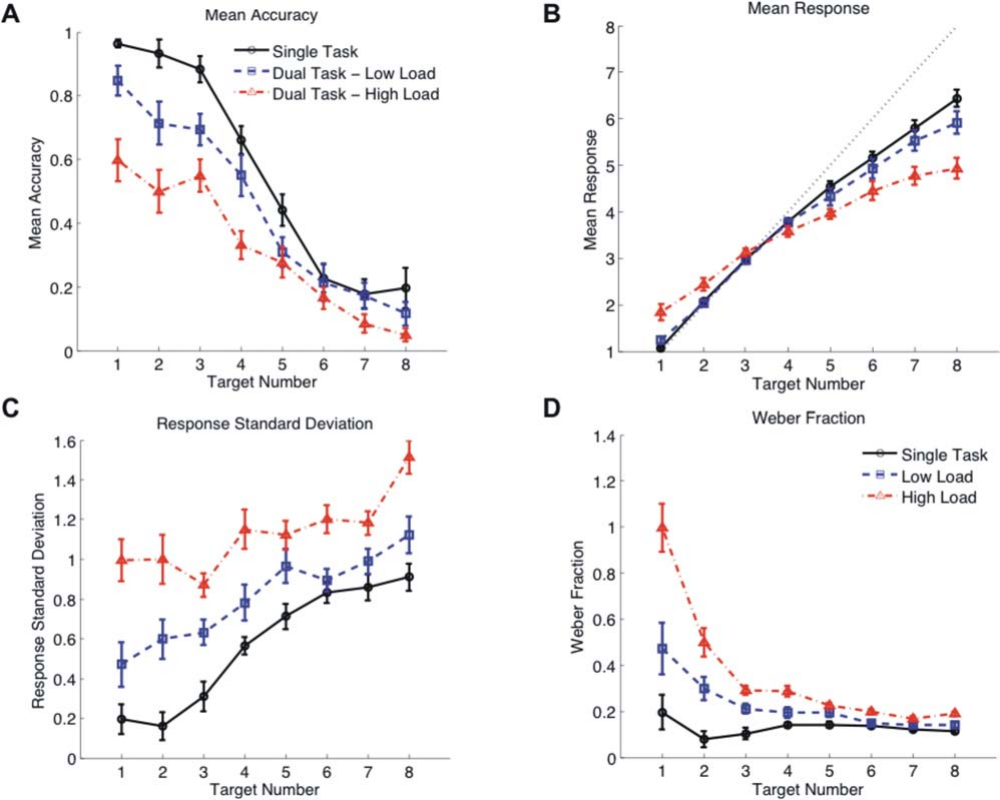
Results of the Numerosity Task. Performance of the numerosity task under the three experimental conditions (single task (black), low load (blue) and high load (red)). Error bars indicate one standard error of the mean. (a) Mean accuracy (proportion correct). (b) Mean responses. The dotted diagonal indicates perfect performance, values above the line represent overestimation, values below underestimation. (c) Response standard deviation. (d) Weber fraction (response STD/target number).

Overall, enumeration accuracy declined steadily with increasing numerosity forming a sigmoidal performance curve (Fig. 3a). We employed a repeated measures ANOVA with within-subject factors “experimental condition” (3 levels: single task, low load, high load) and “target number” (8 levels). There was a significant main effect of condition (*F*(2,26) = 42.49, *p*<.001), with post-hoc comparisons (Bonferroni corrected) showing all three experimental conditions differing significantly from each other. Enumeration accuracy under both dual task conditions was reduced compared to single task condition (single task versus low load: *p* = .004, single task versus high load: *p*<.001). More importantly, enumeration accuracy under high load was more severely impaired than under low load ( *p* = .002).

As expected, enumeration accuracy decreased with increasing target number (*F*(7,91) = 92.65, *p*<.001). There was also a significant interaction between target number and condition (*F*(14,182) = 5.62, *p*<.001), indicating that our attentional manipulation affected subitizing and estimation ranges differently. Therefore, we conducted separate analyses on the subitizing (target number 1–4) and estimation range (target number 5–8). As this study was designed to prevent verbal counting, we refer to larger numerosity judgement as estimation rather than counting.

#### Subitizing range

The main effect of condition was particularly pronounced in the subitizing range. Accuracy dropped from single task conditions to dual task conditions and particularly between low load and high load conditions (main effect: *F*(2,26) = 58.88, *p*<.001; post-hoc comparisons: single task versus low load: *p* = .003, single task versus high load: *p*<.001, low load versus high load: *p*<.001). There was a main effect of target number (*F*(3,39) = 15.94, *p*<.001), but no interaction of target number with condition (*F* (6,78) = 1.29, *p*>.05).

#### Estimation range

Overall, accuracy was low in the estimation range and differences between experimental conditions were less pronounced. Nevertheless, there was a main effect of condition (*F*(2,26) = 5.55, *p* = .010), mainly due to a significant difference between single task and high load condition (post-hoc comparison *p* = .007, all other comparisons: *p*>.05.). As performance reached chance level (12.5%) for numerosities 7 and 8, we repeated the analysis with numerosity range 5–6. There was still a main effect of condition (*F*(2,26) = 3.55, *p = *.043) but post-hoc comparisons did not reach significance.

#### Mean responses, response standard deviation and Weber fraction

Accuracy reflects subject's behaviour only in a binary manner (whether subjects hit exactly the right numerosity or not), but does not consider trials with near misses. We therefore analysed the mean responses given for each target number and their standard deviation as a measure of deviation from the correct response and the distribution of responses. As a measure of discriminability, we adopted the notion of Weber fraction which we define as response standard deviation divided by target number.

#### Mean responses (Fig. 3b)

Comparison of the mean responses to the respective correct target number in each experimental condition (using one-sample t-tests) showed that subjects overestimated the small numerosities (numerosity 1: single task: *t*(13) = 2.48, *p* = .028, low load: *t*(13) = 3.51, *p* = .004, high load: *t*(13) = 4.71, *p*<.001, numerosity 2: high load: *t*(13) = 3.23, *p* = .007, other conditions *p*>.05) and underestimated the larger numerosities from 4 onwards (*t*(13)≤−2.7, *p*≤.018 in all experimental conditions).

A repeated measures ANOVA showed a significant main effect of condition (*F*(2,26) = 9.59, *p* = .001), due to a difference between the single task and the high load condition (post-hoc comparison: *p* = .003, other comparisons: *p*>.05). There was also a significant interaction of condition with target number (*F*(14,182) = 26.67, *p*<.001). Thus, overestimation in the low numerosities and underestimation in the high numerosities occurred more strongly in the high load condition than in all other conditions.

#### Response standard deviation (Fig. 3c)

Response standard deviation increased significantly from single task to low load to high load conditions (main effect: *F*(2,26) = 98.43, *p*<.001, all post-hoc comparisons: *p*<.001). Standard deviations also increased with numerosity (*F*(7,91) = 24.57, *p*<.001) and this effect interacted with the effect of condition (*F*(14,182) = 4.57, *p*<.001).

#### Weber fraction (Fig. 3d)

Weber fraction was consistently higher under dual than under single task conditions, and again higher under high than under low attentional load (main effect: *F*(2,26) = 84.48, *p*<.001; all post-hoc comparisons *p*<.001). All effects replicated when subitizing and estimation ranges were analysed separately (all *p*<.001).

In the single task condition, Weber fraction did not differ across numerosities (*F*(7,91) = 1.46, *p*>.05) consistent with the findings of Ross [25]. Under dual task conditions, however, Weber fraction was highest in the low numerosities and decreased towards higher numerosities (*F*(7,91) = 30.14, *p*<.001). Considering all three conditions, the effect of target number interacted with condition (*F*(14,182) 27.01, *p*<.001). This result confirmed the detrimental effect of attentional load, particularly in the subitizing range, as observed in the accuracy data.

Taken together, these additional analyses showed a clear effect of attentional load also in the higher numerosities. High attentional load resulted in an increase of underestimation, response standard deviation and Weber fraction in the estimation range.

## Discussion

The idea of pre-attentive processing implies that some features of a visual scene are analysed in a privileged manner: unconstrained by a perceptual capacity limit, independent of the number of items to be processed and with the ability to consider the entire visual scene at once [8], [21]. Sagi and Julesz proposed such privileged processing stage for the case of subitizing [9]. Based on this approach, we tested subitizing ability under conditions of reduced attentional resources. We predicted that subitizing should not be affected by dual-task conditions nor by attentional load if it was a truly pre-attentive task. Our results clearly fail to support this prediction. Subitizing accuracy was impaired under dual-task conditions compared to single task conditions, even if the additional task comprised only the detection of a single salient feature (the colour red). More crucially, however, subitizing was even more severely impaired when the additional task required a judgement of high attentional load (a conjunction detection). Thus, the more attentional processing resources were taken away from the numerosity judgement task, the more subitizing ability deteriorated. Weber fractions strikingly mirror the accuracy data, indicating that discrimination ability decreased dramatically under high attentional load particularly in the subitizing range. These results challenge the traditional notion of a pre-attentive subitizing mechanism as proposed by many previous studies [3], [4], [6], [9].

Our results are in line with recent works demonstrating an impairment of subitizing performance in the attentional blink [22], [23] and under conditions of inattentional blindness [24]. In addition to these studies, however, we demonstrate a differential effect on subitizing performance depending on the amount of attentional resources that are drawn away from the enumeration task.

Furthermore, we also found a clear effect of dual task conditions and attentional load in the estimation range (numerosities 5–8), apparent as an increase in the degree of underestimation and response standard deviation. These findings suggest that the withdrawal of attentional resources affect numerosity judgement in a systematic manner: the more processing resources are taken away and the more difficult numerosity judgement becomes at higher numerosities, the more performance deviates from an unaffected distribution.

The fact that both the enumeration of small and large quantities is equally affected by the manipulation of attentional resources (in proportion to their respective difficulty) could be interpreted as evidence against a functional dichotomy between subitizing and counting. Our results suggest that both small and large numerosity judgment reflect stages on a single, continuous enumeration mechanism. However, this study was not designed to investigate the nature of these mechanisms, and more specific studies are needed to address this issue. Nevertheless, our results render one of the main arguments for such a dichotomy unlikely: that subitizing is parallel and pre-attentive and might therefore be different from an attentive counting or estimation stage. In support of a continuous enumeration mechanism, Ross showed that Weber fractions are consistently around 25% across a wide range of numerosities, which implies that numerosity judgements in the subitizing range always fall within the performance limit set by this Weber fraction [25]. This finding provides a simple explanation for why subitizing appears relatively effortless and further strengthens the idea that numerosity judgement is subserved by a single mechanism rather than two functionally separate ones. Our results confirm those of Ross: in the single task condition, Weber fraction was constant across all numerosities and attentional load affected Weber fractions without any sharp discontinuity between the subitizing and the estimation range. Thus, our findings and those of Ross [25] raise the possibility that previous reports of preserved subitizing ability in neglect patients [10] as well as differential brain activations in healthy subjects [4], [6] might reflect a quantitative rather than qualitative difference between enumerating small and large numerosities.
